# A Case of Lithotomy, Attended with Some Remarkable Circumstances

**Published:** 1800

**Authors:** William Wickham

**Affiliations:** Surgeon of the Winchester Hospital.


VIII. *A Case of Lithotomy, attended with some remarkable Circumstances. By Mr.* William Wickham, *Surgeon of the Winchester Hospital. Communicated in a Letter to* John Latham, *M. D. F. R. S. Physician at Romsey, in Hampshire; and by him to Dr.* Simmons.

THOMAS STEVENSON, aged nine years, was admitted into the Winchester Hospital, June 21, 1797, as having a stone in the bladder, to undergo the operation for extracting the same. He was examined by the gentleman whose care he fell under, and a stone being evidently discovered, the operation was determined upon, and on the 10th day of July we met for the purpose of performing it. In passing the staff, a degree of resistance at the neck of the bladder was met with, sufficient to prevent its entry into the bladder, notwithstanding which, the stone could be distinctly felt at the point of the instrument. On examination, by the finger, up the rectum, the stone could not be felt; but it was evident that the staff was not sufficiently in the bladder to allow us to perform perform the operation. Frequent attempts were now made to prefs it into the bladder, all of which gave extreme pain, and caufed a great deal of ftraining and forcing. Different fized ftaffs were tried but with no better fuccefs; under the idea that fome ftricture, or fpafmodic affection, of the neck of the bladder, might be the caufe of this obftruction to the ftaff, the patient was fent back to his bed, and we directed for him aperient medicines, opiates, &c.

In about a fortnight the parties again met to perform the operation, but the fame difficulty occurred as before, and the patient expreffed great pain when the ftaff got as far as the obftruction. I paffed myfelf, at this examination, a very fmall ftaff perfectly into the bladder, with a tolerable degree of eafe, and without giving much pain; but the ftaff was of fuch length and curvature, as made it impoffible to operate with it without running a very great rifk. The patient was again returned to his bed, and the fame lenient treatment obferved as formerly, with the addition of camphor and the warm bath. A few days after this we met again, having directed him to be put into a warm bath, and an opiate to be given to him immediately before the meeting; an attempt was then made to paſs the ſtaff while he was in the bath, but without ſucceſs. He was then taken out, and put upon the table, where ſtaffs of every ſize, and every means that could be thought of, were tried, but all failed; though the ſtone was diſtinctly felt at the end of the ſtaff. He was again returned to his bed while we could ſend to our Inſtrument-maker in London, for a *very ſmall ſtaff*, of the length and curvature requiſite for the ſize of the patient; but immediately after this, fever, with great tenſion of the whole abdomen, and ſuch a degree of inflammation took place, as to prevent any further attempts in the way of operation. He died on the 3d of September, a month after the laſt examination.

On opening the body, we found that a great degree of inflammation had ſpread itſelf over the lower part of the abdomen, and occaſioned ſtrong adheſions between the inteſtines and peritoneum. A conſiderable quantity of matter was diſcovered upon the left pſoas muſcle, and an ulceration of the ſize of a ſhilling had taken place in that part of the rectum which is nearly attached to the bladder.

Upon taking out the bladder and opening it, no particular difeafe appeared within; fome inflammation, of courfe, was vifible, but in a much lefs degree than externally. The ftone, which weighed three drachms, was of the nature of the fufible calculus, as defcribed by Dr. Wollafton in his Analyfis of Gouty and Urinary Concretions.* It did not adhere to any part, nor was it fixed in the neck of the bladder; it is true, before the bladder was taken out, upon preffing the fundus and body, no ftone could be felt, fo that it might have been at that time in or near to the neck of the bladder, but certainly it was not fixed there; when the neck of the bladder was divided in taking it out, the ftone was found in the middle of the bladder.

I have annexed a drawing of the ftone, accurately finifhed, (See Fig. 7. Plate II.) which will fhow the poffibility of the fmall end being preffed into the neck of the bladder, fo as to have prevented a ftaff from forcing it back; I think it probable that the contraction of the bladder might be fo great as to prefs the ftone in the fituation I fuppofe it was in (partly in the

* Philof. Tranf. for 1797, Part II. page 390.

 neck neck of the bladder) particularly as the patient was always in great forcing pains at the time of preſſing the ſtone with the ſtaff; and by the neck of the ſtone, it is probable, ſuch a degree of contraction upon that part might have taken place, as to prevent its returning by the preſſure made by the ſtaff upon the ſmall end.

This caſe ſhows us the neceſſity of ſometimes deviating from the uſual directions given in Lithotomy; and the probability of ſucceſs, had the operation been attempted in the manner Mr. Abernethy * performed it, by cutting upon the end of the ſtaff, and paſſing a probe-pointed knife by the ſide of the ſtone, and then extracting by the forceps. It is likewiſe ſimilar to a caſe that occurred to Mr. Earle, † with this difference only, that the ſtone in his caſe was confined there by a fungus.

*Wincheſter,*

*January* 10, 1798.

* See Mr. Earle's Obſ. on the Operation for the Stone. 8vo. 1793. page 75.

† Ibid. page 71.

